# Association between Glucocorticoid-Induced Osteoporosis and Myasthenia Gravis: A Cross-Sectional Study

**DOI:** 10.1371/journal.pone.0126579

**Published:** 2015-05-12

**Authors:** Shingo Konno, Shigeaki Suzuki, Masayuki Masuda, Yuriko Nagane, Emiko Tsuda, Hiroyuki Murai, Tomihiro Imai, Toshiki Fujioka, Norihiro Suzuki, Kimiaki Utsugisawa

**Affiliations:** 1 Department of Neurology, Toho University Ohashi Medical Center, Tokyo, Japan; 2 Department of Neurology, Keio University School of Medicine, Tokyo, Japan; 3 Department of Neurology, Tokyo Medical University, Tokyo, Japan; 4 Department of Neurology, Hanamaki General Hospital, Iwate, Japan; 5 Department of Neurology, Sapporo Medical University, Hokkaido, Japan; 6 Department of Neurology, Neurological Institute, Graduate School of Medical Sciences, Kyushu University, Fukuoka, Japan; Istanbul University, TURKEY

## Abstract

**Purpose:**

To investigate the association between glucocorticoid-induced osteoporosis and myasthenia gravis (MG) using a cross-sectional survey in Japan.

**Methods:**

We studied 363 patients with MG (female 68%; mean age, 57 ± 16 years) who were followed at six Japanese centers between April and July 2012. We evaluated the clinical information of MG and fractures, bone markers, and radiological assessment. Quality of life was measured using an MG-specific battery, MG-QOL15.

**Results:**

Glucocorticoids were administered in 283 (78%) of 363 MG patients. Eighteen (6%) of 283 MG patients treated with prednisolone had a history of osteoporotic fractures. The duration of glucocorticoid therapy, but not the dose of prednisolone, was associated with the osteoporotic fractures in MG patients. Bone mineral density was significantly decreased in the MG patients with fractures. The multivariate analyses showed that the total quantitative MG score was the only independent factor associated with osteoporotic fractures (OR = 1.30, 95% CI 1.02–1.67, p = 0.03). MG patients who had experienced fractures reported more severe difficulties in activities of daily living.

**Conclusion:**

Glucocorticoid-induced osteoporosis aggravates quality of life in patients with MG.

## Introduction

Glucocorticoids are widely used to treat a variety of systemic autoimmune diseases. Although glucocorticoids are effective, various side effects of long-term treatment, such as osteoporosis, represent an important problem. The risk of glucocorticoid-induced osteoporosis appears to be similar in men and women, and consequent osteoporotic fractures and impairments of activities of daily living result in huge economic losses at the national level [1]. Patients with autoimmune disease may have a high risk of osteoporotic fracture. A meta-analysis revealed bone mineral density loss after long-term treatment with low-dose glucocorticoids in patients with rheumatoid arthritis [2]. In addition, the cumulative glucocorticoids used in the treatment of systemic lupus erythematosus have been significantly associated with the development of osteoporotic fractures [3].

Myasthenia gravis (MG) is the most common autoimmune neuromuscular disorder and is mediated by autoantibodies to acetylcholine receptor or muscle-specific tyrosine kinase [4]. Although advances in immunosuppressive treatment have dramatically reduced the mortality of MG, many MG patients still find it difficult to maintain their daily activities due to the long-term side effects of glucocorticoids. However, little attention has been paid to the impact of glucocorticoid-induced osteoporosis on the activities of MG patients. We hypothesized that fracture associated with the use of glucocorticoids would have very deleterious effects on the quality of life of MG patients.

The purpose of the present study was to investigate the association between glucocorticoid-induced osteoporosis and MG using a cross-sectional survey of six centers in Japan.

## Patients and Methods

### Patients

We evaluated patients with established MG who were followed at Toho University Ohashi Medical Center, Keio University Hospital, Tokyo Medical University Hospital, Hanamaki General Hospital, Sapporo Medical University Hospital, and Iizuka Hospital between April and July 2012. To avoid potential bias, we enrolled consecutive patients with various stages of illness over a short duration in this multicenter, cross-sectional study. We studied 363 MG patients (M:F = 116:247; mean age, 56.5 ± 15.9 years) who underwent bone-related laboratory and radiological assessment in addition to complete clinical examination.

All clinical information was collected after the patients provided written informed consent. All study protocols were specifically approved by the institutional review board of the ethics committee of Toho University Ohashi Medical Center, the ethics committee of Keio University Hospital, the ethics committee of Tokyo Medical University Hospital, the ethics committee of Hanamaki General Hospital, the ethics committee of Sapporo Medical University Hospital, and the ethics committee of Kyushu University School of Medicine. These clinical investigations have been conducted according to the principles expressed in the Declaration of Helsinki.

### Methods

The diagnosis of MG was based on clinical findings (fluctuating symptoms with easy fatigability and recovery after rest) along with amelioration of symptoms after an intravenous administration of acetylcholinesterase inhibitors, decremental muscle response to a train of low-frequency repetitive nerve stimuli, or the presence of autoantibody against skeletal muscle acetylcholine receptors or muscle-specific tyrosine kinases [4]. Clinical status and severity were determined by the recommendations of the Myasthenia Gravis Foundation of America [5]. In Japan, prednisolone is generally used for MG treatment. The disease-specific quality of life was evaluated with the Japanese translated version of the MG-QOL15 [6], a self-appraised scoring system. This tool is simple, easy to administer, and user-friendly, and provides a quick assessment of the influence of a disease.

Bone mineral density at the lumbar spine (L2-L4) and femoral neck was measured using a dual-energy X-ray absorptiometry system provided by Hologic (Waltham, MA) or GE-Healthcare (Madison, WI). Levels of serum pyridinoline cross-linked amino-terminal telopeptide of type I collagen (NTx) and bone isoform of alkaline phosphatase (BAP) levels were also measured.

Comparisons of clinical and laboratory characteristics between MG patients with and without fractures were performed using the chi-squared or Mann-Whitney U-test as appropriate. Values of p<0.05 were considered statistically significant. Statistical analyses were performed using JMP version 9 statistical software (SAS Institute Inc., Cary, NC).

## Results

Of 363 MG patients, 283 (78%) had been treated with prednisolone at a dose of ≥ 5 mg/day for three months or more. Glucocorticoid-induced osteoporosis was evaluated based on the development of fracture after MG onset. Symptomatic fractures were developed in 19 (5%) patients of the 363 MG patients. Although a history of spine, arms, hips, or wrists fractures was seen in only 1 (1%) of the 80 MG patients without glucocorticoid treatment, 18 (6%) of 283 MG patients treated with glucocorticoids had one or more such fractures.

We divided the 283 MG patients treated with prednisolone into two groups: MG patients with fractures (n = 18) and those without (n = 265). The clinical and laboratory features of the two groups were compared (Table 1). There were no differences in gender or age. The disease duration of MG was longer in the MG patients with fractures than in those without (16.7 ± 14.1 vs. 10.9 ± 9.5 years, p = 0.048). When MG subtype was divided into early-onset, late-onset, and thymoma-associated, there were no differences between the fracture and non-fracture groups. Because a remission or minimal manifestation status is usually regarded as the goal of MG treatment, we determined the number of patients who reached this goal. Of the 283 MG patients treated with prednisolone, 123 (43%) achieved a remission or minimal manifestation status. There was again no difference in favorable MG status between the fracture and non-fracture groups. In contrast, the total quantitative MG (QMG) score was significantly higher in the MG patients with fractures than in those without fractures (22.1 ± 13.3 vs. 6.8 ± 4.8, p < 0.0001). Thus, the MG patients who had experienced osteoporotic fractures suffered from severe MG symptoms.

**Table 1:** Characteristics of 283 patients with myasthenia gravis (MG) treated with prednisolone

	With fractures (n = 18)	Without fractures (n = 265)	P
Gender (M:F)	5:13	86:179	0.9
Age (yr)	61.5 ± 15.2	55.4 ± 15.9	0.1
Disease duration (yr)	16.7 ± 14.1	10.9 ± 9.5	0.048*
MG subtype			
Early-onset	9 (50%)	121(46%)	0.7
Late-onset	5 (28%)	72 (27%)	1
Thymoma-associated	4 (22%)	72 (27%)	0.7
MG status			
Minimal manifestation or better	5 (28%)	118 (45%)	0.2
Total quantitative MG score	22.1 ± 13.3	6.8 ± 4.8	<0.0001*
Prednisolone treatment			
Maximum dose (mg/d)	39.7 ± 21.1	30.6 ± 19.0	0.08
Current dose (mg/d)	8.7 ± 7.4	6.0 ± 6.4	0.08
1-year total dose (mg)	2977 ± 2456	2044 ± 1862	0.1
Duration of treatment (yr)	12.1 ± 10.4	6.4 ± 6.2	0.02*
Risk factors			
Body mass index	23.8 ± 4.7	22.8 ± 3.7	0.6
Parental hip fracture	0	7 (3%)	0.9
Current smoking	3 (16%)	33 (12%)	0.8
Excess of alcohol intake	1 (6%)	11 (4%)	0.8
Rheumatoid arthritis	0	3 (1%)	0.5
Bone mineral density			
Femoral neck T score	−2.0 ± 1.3	−1.1 ± 1.1	0.003*
Lumbar spine T score	−1.9 ± 1.2 (n = 14)	−0.9 ± 1.6 (n = 180)	0.3
Bone markers			
Serum NTx (mmolBEC/L)	15.5 ± 4.2	13.9 ± 6.3	0.04*
Serum BAP (μg/L)	12.8 ± 4.8	11.8 ± 6.0	0.1
Treatment			
Vitamin D	4 (22%)	71 (27%)	0.6
Calcium	1 (6%)	24 (9%)	0.6
Bisphosphonates	11(61%)	135 (51%)	0.4

*statistically significant

NTX, pyridinoline cross-linked amino-terminal telopeptide of type I collagen

BAP, bone isoform of alkaline phosphatase

We next compared the data on prednisolone treatment. Maximum dose, current dose, and 1-year total dose of prednisolone tended to be higher in the MG patients with fractures than in those without, but significant differences were not found. However, the duration of prednisolone treatment was significantly longer in the MG patients with fractures than in those without (12.1 ± 10.4 vs. 6.4 ± 6.2 years, p = 0.02).

Other risk factors for osteoporotic facture were also investigated. There were no differences in body mass index, parental history of hip fracture, current smoking, alcohol intake of three or more units daily intake, and rheumatoid arthritis between the two groups.

We simultaneously measured the bone mineral density at the lumbar spine and femoral neck. The femoral neck T score of the patients with fractures was significantly lower than that in the patients without fractures (−2.0 ± 1.3 vs. −1.1 ± 1.1, p = 0.003). The lumbar spine T score also showed a similar tendency. With regard to the bone markers, there were no differences of serum BAP levels between the two groups. Serum NTx levels were significantly higher in the MG patients with fractures than in those without fractures (15.5 ± 4.2 vs. 13.9 ± 6.3 mmol bone collagen equivalent/L, p = 0.04). In addition to calcium or vitamin D supplementation, bisphosphonates were administered in 146 (52%) of the 283 MG patients treated by prednisolone. There was no difference in the frequency of bisphosphonate use between the two groups. Teriparatide and denosumab were not used.

We next examined the clinical or laboratory factors strongly associated with the development of osteoporotic fractures in MG patients treated with prednisolone. The multivariate analyses revealed that the total QMG score was the only independent factor associated with osteoporotic fractures (OR = 1.30, 95% CI 1.02–1.67, p = 0.03). Since the total QMG score may be potentially influenced by the time of the day, we consider the possibility that the severity of MG is not accurately evaluated by total QMG score. So we additionally performed the multivariate analysis excluding the total QMG score from the clinical and laboratory factors. There was no statistically significant factor associated with osteoporotic fractures.

**Fig 1:**
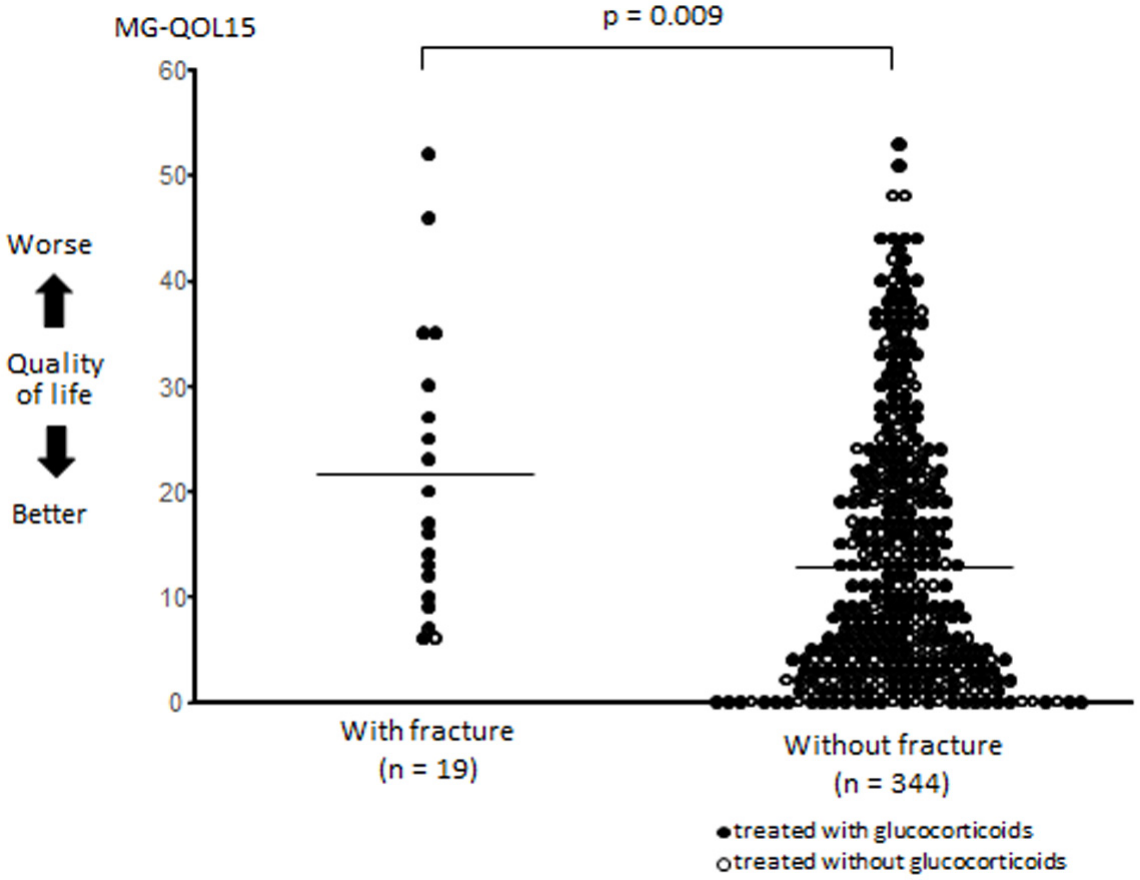
Quality of life was compared between myasthenia gravis (MG) patients with or without fractures using MG-QOL15 scores. The bar indicates the average.

We also investigated the impact of fracture on the quality of life in MG patients with or without glucocorticoids. The MG-QOL15, which is useful for identifying satisfaction or dissatisfaction with the manifestations of MG, can capture various aspects of quality of life [6]. As shown in Fig 1, the total MG-QOL15 score was significantly higher in the 19 MG patients with fractures than in the 344 MG patients without fractures (21.2 ± 13.4 vs. 13.9 ± 12.5, p = 0.009). MG patients who had experienced fractures reported greater difficulties in their activities of daily living.

Finally, we calculated the 10-year probability of major osteoporotic fracture in our patients using the Fracture Risk Assessment Tool (FRAX). The probability increased progressively with increasing dose of glucocorticoids with the proper adjustment of FRAX [7]. We found that the risk of osteoporotic fracture exceeded 12% in female MG patients in the 6th decade of age.

## Discussion

The present multicenter, cross-sectional survey in Japan investigated the clinical significance of glucocorticoid-induced osteoporosis in 363 patients with MG. The main results can be summarized as follows: (i) osteoporotic fractures were developed in 18 (6%) of 283 MG patients treated with glucocorticoids, (ii) the total QMG score was the only independent factor associated with osteoporotic fractures, and (iii) osteoporotic fractures made the quality of life of MG patients significantly worse. We think it is difficult to discriminate whether the dissatisfaction evaluated by the MG-QOL15 is due to osteoporotic fractures or uncontrolled MG condition. However, we believe the prevention of osteoporotic fracture is an important goal of management of MG patients.

A retrospective cohort study using the UK General Practice Research Database revealed that overall risk of fracture in patients with MG was not statistically increased compared with age- and gender-matched controls irrespective of glucocorticoid use [8]. In contrast, the population-based retrospective cohort study from the Taiwan National Health Insurance Research database provided evidence that MG was associated with a high risk of osteoporosis regardless of glucocorticoid use [9]. Although these two large population-based studies were conducted using a similar cohort approach, their conclusions were completely at odds. The discrepancy was explained by the differences in average age of the included MG patients: 41 years in the Taiwanese cohort vs. 60 years in the English cohort [9].

The strength of the present study is that we collected the comprehensive clinical and laboratory data on both MG and glucocorticoid-induced osteoporosis from the individual patients. We observed that the duration of glucocorticoid therapy, but not the dose of prednisolone, was associated with development of osteoporotic fractures in MG patients. However, the multivariate analyses revealed the duration of glucocorticoid therapy was not the independent factor associated with osteoporotic fractures. We think the total dose of prednisolone for the entire period may be more important in the development of glucocorticoid-induced osteoporosis more than the duration of prednisolone treatment. Since we could not accurately calculate the accumulated dose of prednisolone, we did not include the total dose of prednisolone among clinical factors.

The striking feature of our study was that the severity of MG was the most important factor influencing the development of glucocorticoid-induced osteoporosis. We speculate that MG symptoms may predispose a patient to develop osteoporotic fractures. The association is likely ascribable to various factors, including physical inactivity caused by muscle weakness, lack of outdoor activity, and decreased exposure to sunlight due to disability [9]. In addition to limb weakness, impaired vision influenced by ptosis and/or diplopia contributes to the risk of falls in patients with MG. Moreover, we clearly demonstrated for the first time that the osteoporotic fractures had harmful effects on the daily activities of MG patients.

The fracture risk caused by glucocorticoid-induced osteoporosis is not estimated accurately based on the Fracture Risk Assessment Tool (FRAX). However, we calculated the 10-year probability of major osteoporotic fracture with the proper adjustment of FRAX [7]. We found that the high risk of osteoporotic fracture in female older MG patients. In keeping with this result, a previous study found that the frequency of MG onset in individuals over 60 years is markedly increased in both Asian and Caucasian MG patients [4]. Taking these results together, we consider that special attention should be paid to the prevention of glucocorticoid-induced bone disease in older patients with MG.

Bisphosphonates are considered to be the first-line options for the treatment of glucocorticoid-induced osteoporosis [1]. In fact, 52% of our MG patients were treated with bisphosphonates. Wakata et al. commented the MG patients treated with glucocorticoids had an acceptable risk of bone loss if prophylactic medication was administered [10]. However, our findings suggest that effective strategies are required for better management of MG patients. In this regard, bisphosphonates appear to be less effective in the protection of bone mineral density in patients with glucocorticoid-induced osteoporosis than they are in patients with other forms of osteoporosis [1]. Treatment with teriparatide or denosumab is expected to produce better outcomes in the management of MG.

Several limitations of the present study bear mention. First, we retrospectively collected the clinical information from MG patients. Data from the time of development of osteoporotic fracture were not available. Second, we did not examine routine X-rays in order to detect the asymptomatic fractures. Thus the frequency of glucocorticoid-induced osteoporosis may have been underestimated. Despite these limitations, however, we demonstrated the tight association between glucocorticoid-induced osteoporosis and MG.

In conclusion, glucocorticoid-induced osteoporosis aggravates the quality of life of patients with MG. To avoid osteoporotic fracture, neurologists have to consider to shorten the duration of glucocorticoids treatment for MG.

## Supporting Information

S1 Table The whole dataset (n = 363) subjected to the present analysis. (PDF)
