# Supplementary material for: Association between Glucocorticoid-Induced Osteoporosis and Myasthenia Gravis: A Cross-Sectional Study
Source: PLoS One. 2015 May 12;10(5):e0126579. doi: 10.1371/journal.pone.0126579 (PMC4428766; doi:10.1371/journal.pone.0126579)
Supplement: S1 Table — (PDF) [file pone.0126579.s001.pdf]

Table S1 The whole dataset (n = 363) subjected to the present analysis

ID	Sex	Age	Onset	Duration	Thymoma	MGFA	CSR	PR	MM	I	U	W	BMD	Tscore	BAP	NTx	PSL	PSL(yr)	PSL(dose)	PSL(current)
9	2	42	28	14.5	0	2	0	0	1	0	0	0	0.848	−0.55462185	21	22	1	12	5	2.5
17	2	77	47	29.3	0	3	0	0	0	0	0	1	0.48	−3.64705882	13	13.7	1	16	30	5
19	1	72	46	26.7	1	2	0	0	0	1	0	0	0.834	−0.89230769	7.5	18.6	1	23	40	5
20	2	73	39	31.3	0	2	1	0	0	0	0	0	0.796	−0.99159664	19.3	19.9	0			
25	1	51	35	16.1	1	1	0	0	0	0	0	1	1.011	0.469230769	11.5	12.9	1	5.5	30	8
26	2	83	53	29.2	1	2	0	0	0	0	1	0	0.661	−2.12605042	9.8	11.5	0			
27	2	66	46	20.2	0	2	0	0	0	0	1	0	0.721	−1.62184874	15	22.4	0			
28	1	48	29	18.8	1	2	0	0	0	0	0	1	0.758	−1.47692308	10.6	11.3	1	18	60	20
31	2	64	38	24.8	0	3	0	0	0	1	0	0	0.644	−2.26890756	11.2	19.6	1	7	15	5
36	2	64	43	20.8	1	3	0	0	0	1	0	0	0.539	−3.1512605	10.8	14.6	1	20	30	7
37	1	38	20	18.1	1	3	0	0	0	1	0	0	0.771	−1.37692308	12.8	13.4	1	17.5	60	2.5
43	2	39	24	14.6	0	5	0	0	0	1	0	0	0.661	−2.12605042	7.6	9.9	1	14.5	40	10
54	1	59	33	25.3	1	5	0	0	0	1	0	0	0.746	−1.56923077	13.6	12.4	1	25	60	8
55	2	75	51	23.9	1	2	0	0	0	1	0	0	0.887	−0.22689076	17.4	10.7	0			
65	2	65	45	20	0	2	0	1	0	0	0	0	0.982	0.571428571	11.4	9.3	1	6	20	0
123	2	64	43	20.4	1	5	0	0	0	1	0	0	0.64	−2.30252101	14.2	17.2	1	20	60	8
124	1	60	28	12.1	0	2	0	1	0	0	0	0	1.115	1.269230769	11.2	14	0			
125	2	50	38	11.5	0	2	0	0	0	1	0	0	0.709	−1.72268908	6.9	11.6	0			
129	2	52	26	25.1	0	2	0	1	0	0	0	0	1.009	0.798319328	12.6	15.4	1	25	80	2.5
141	1	67	57	10.7	1	3	0	1	0	0	0	0	1.381	3.315384615	10.6	10.5	1	5	20	2.5
145	2	41	28	10.7	0	2	0	0	1	0	0	0	0.994	0.672268908	5.3	10.4	1	10	60	2.5
149	2	37	1	35.5	0	2	0	0	0	0	1	0	0.777	−1.1512605	5.4	11.5	1	10.5	30	8
161	2	65	17	47.6	0	3	0	0	0	1	0	0	0.685	−1.92436975	7.3	8.9	1	9.5	60	16
165	2	78	68	9.6	0	4	0	0	1	0	0	0	0.719	−1.63865546	12.8	18.6	1	6	30	0
175	1	59	48	10.7	0	2	0	0	0	0	1	0	0.695	−1.96153846	19.9	10.4	1	5	10	7
200	2	61	52	9.3	1	3	0	0	1	0	0	0	0.737	−1.48739496	14.8	12.5	1	6	20	5
201	2	33	24	8.6	0	3	0	0	0	0	1	0	0.782	−1.1092437	10.9	9.2	0			
206	1	63	57	6.4	0	2	0	0	0	1	0	0	0.673	−2.13076923	9	9.3	1	1.2	10	5
210	1	53	45	7.5	0	2	0	0	1	0	0	0	0.791	−1.22307692	14.7	26.5	1	0.2	20	0
218	2	71	66	5.3	1	3	0	0	0	1	0	0	0.831	−0.69747899	11.5	9.6	1	5.3	20	8
231	1	75	70	4.4	0	1	0	0	1	0	0	0	0.758	−1.47692308	16.5	14.1	0			
239	2	44	40	4.6	1	3	0	0	1	0	0	0	0.987	0.613445378	9.7	12.3	1	4.5	25	10
243	1	53	26	27	0	2	0	0	1	0	0	0	0.712	−1.83076923	7.8	8.1	1	11	30	15
247	2	28	23	4.4	0	2	0	0	0	1	0	0	0.983	0.579831933	7	14.6	1	1.4	10	10
249	2	32	19	12	0	2	0	0	0	1	0	0	0.789	−1.05042017	14.9	11.1	1	12	50	15
252	2	58	52	5.8	0	1	0	0	1	0	0	0	0.749	−1.38655462	13.6	17.4	0			
256	2	50	46	3.5	1	5	0	0	0	1	0	0	0.79	−1.04201681	6.6	13.1	1	3	40	10
257	1	63	59	3.6	1	3	0	1	0	0	0	0	0.864	−0.66153846	8.1	11.7	1	3.5	20	5
259	1	77	74	3.6	0	1	0	0	1	0	0	0	0.765	−1.42307692	10.2	11.2	0			
261	2	35	26	8.6	0	4	0	1	0	0	0	0	0.72	−1.6302521	11	12.9	1	4	40	7
262	2	42	38	4.2	0	2	0	0	0	0	0	1	0.794	−1.00840336	8	26.8	0			
264	2	30	27	3.5	0	2	0	0	1	0	0	0	0.751	−1.3697479	6.3	19	1	3	20	10
276	1	64	61	2.8	1	3	0	0	0	0	0	0	0.736	−1.64615385	8.6	12.8	1	2.7	10	6
277	2	55	52	2.8	0	1	0	0	1	0	0	0	0.626	−2.42016807	13.6	14.6	0			
279	1	30	27	2.7	0	3	0	0	0	0	0	0	0.952	0.015384615	10.7	11.6	1	2.6	20	15
280	2	43	24	19	0	2	0	0	0	1	0	0	0.873	−0.34453782	5.4	13.9	1	3.5	30	0
282	2	61	58	2.5	1	2	0	0	1	0	0	0	0.75	−1.37815126	10.1	15.8	1	2	10	7
287	2	65	63	2.1	1	5	0	0	0	1	0	0	0.61	−2.55462185	7.4	13.5	1	1.7	40	6
295	1	56	54	2	0	2	0	0	0	1	0	0	0.981	0.238461538	13.2	11.2	1	1	10	6
299	2	75	73	2	0	1	0	0	0	1	0	0	0.704	−1.76470588	9.7	11.2	0			
302	2	46	43	2.2	0	3	0	1	0	0	0	0	0.845	−0.57983193	8.1	11.6	1	1.8	25	7
315	2	41	40	0.9	1	4	0	0	1	0	0	0	0.946	0.268907563	11.4	12.8	1	0.8	20	12
316	2	29	26	3.6	0	3	0	0	0	1	0	0	0.872	−0.35294118	3.9	11.5	1	3.5	20	18
317	2	35	34	1.2	0	3	0	0	0	0	1	0	0.817	−0.81512605	4.7	14.9	1	1	20	20
319	2	51	31	20	0	3	0	0	0	1	0	0	1.093	1.504201681	6.8	15.7	1	20	15	15
320	2	40	38	1.7	0	2	0	0	0	1	0	0	0.969	0.462184874	12	10.6	1	0.6	10	6

324	2	39	24	15	0	5	0	0	0	1	0	0	0.822	−0.77310924	10.5	8.6	1	15	80	30
328	2	29	28	0.8	1	1	0	0	1	0	0	0	0.92	1.220183486	9.9	9.9	1	0.5	10	8
1006	2	75	67	8	1	5	0	0	1	0	0	0	0.689	−0.89908257	8.9	10.6	1	8	20	2.5
1008	2	60	51	9	1	2	0	0	1	0	0	0	0.657	−1.19266055	12.5	11	1	9	10	5
1011	2	53	35	17	0	3	0	0	0	1	0	0	0.52	−2.44954128	36.7	80	1	15	64	6
1017	1	41	28	12	0	2	0	1	0	0	0	0	0.595	−2.16129032	16.6	12.4	1	9	10	5
1032	2	32	7	24	0	2	0	1	0	0	0	0	0.656	−1.20183486	8.9	11.9	1	23	10	2.5
1034	1	78	66	11	1	2	0	1	0	0	0	0	0.782	−0.65322581	12.1	9.5	1	8	10	5
1043	2	33	22	11	0	2	0	1	0	0	0	0	0.556	−2.11926606	8	10.2	1	8	10	5
1046	2	47	38	9	0	2	0	0	0	1	0	0	0.701	−0.78899083	7.7	15.2	1	4	15	0
1054	2	55	48	7	1	5	0	0	0	1	0	0	0.679	−0.99082569	11.6	20.5	1	7	5	5
1055	2	61	51	10	0	3	0	0	1	0	0	0	0.578	−1.91743119	8.3	9.6	1	6	10	5
1056	2	67	54	13	0	2	0	0	1	0	0	0	0.764	−0.21100917	6.1	8.1	1	8	10	5
1063	1	30	19	11	0	3	0	0	0	1	0	0	0.597	−2.14516129	11.4	9.1	1	7	40	7.5
1066	2	58	46	11	0	3	0	0	1	0	0	0	0.655	−1.21100917	10.2	7.4	1	10	60	5
1071	2	25	17	7	0	2	0	1	0	0	0	0	0.646	−1.29357798	11.2	14.5	1	7	10	5
1074	1	65	58	7	0	1	0	0	0	1	0	0	0.836	−0.21774194	16.9	13.6	1	6	10	5
1079	2	45	36	9	0	2	0	0	1	0	0	0	0.791	0.036697248	21.4	13.8	1	7	10	0
1083	2	59	50	9	0	1	0	0	0	1	0	0	0.661	−1.1559633	13.3	11.2	1	6	10	2.5
1087	2	61	56	5	1	2	0	1	0	0	0	0	0.737	−0.4587156	13.4	11.6	1	4	5	0
1088	2	17	8	9	0	2	0	0	0	1	0	0	0.556	−2.11926606	16.5	13.1	1	5	10	10
1091	2	38	28	9	1	2	0	1	0	0	0	0	0.675	−1.02752294	15	10.5	1	9	10	5
1092	1	63	55	7	0	2	0	1	0	0	0	0	0.971	0.870967742	19.6	11.7	1	5	10	5
1094	2	65	57	7	0	2	0	0	1	0	0	0	0.487	−2.75229358	24.5	16.7	1	4	10	5
1095	1	52	45	7	0	3	0	0	0	1	0	0	0.63	−1.87903226	7.6	17.6	1	2	10	0
1098	2	52	45	7	0	3	0	0	0	0	1	0	0.694	−0.85321101	8.8	13.1	1	4	10	10
1100	2	40	33	7	0	2	0	1	0	0	0	0	0.68	−0.98165138	10.9	9.6	1	6	10	2.5
1101	2	70	44	25	1	5	0	0	0	1	0	0	0.477	−2.8440367	12	12.8	1	25	60	5
1103	2	23	17	6	0	3	0	1	0	0	0	0	0.746	−0.37614679	14.6	11.3	1	2.5	10	0
1107	2	71	64	6	0	1	0	0	1	0	0	0	0.565	−2.03669725	23.6	13.6	1	1.3	5	0
1111	2	76	74	2	0	2	0	1	0	0	0	0	0.483	−2.78899083	16.5	13.2	1	1.5	5	0
1112	1	16	9	7	0	2	0	1	0	0	0	0	0.744	−0.95967742	49.2	34.2	1	4	10	5
1113	2	68	63	5	0	2	0	0	0	1	0	0	0.552	−2.1559633	8.1	13.4	1	4	5	5
1114	2	57	50	7	0	3	0	0	0	1	0	0	0.724	−0.57798165	9.3	8.5	1	5	10	10
1116	2	34	29	5	0	3	0	0	0	1	0	0	0.816	0.266055046	20.4	9.2	1	2	20	0
1119	2	84	79	5	0	3	0	0	1	0	0	0	0.501	−2.62385321	15.4	16.3	1	4	10	5
1122	2	47	44	3	1	5	0	1	0	0	0	0	0.819	0.293577982	19.3	13.8	1	1.5	15	0
1123	2	73	67	6	0	2	0	1	0	0	0	0	0.538	−2.28440367	12.2	14.9	0			
1124	2	68	61	7	0	2	0	0	1	0	0	0	0.675	−1.02752294	19.5	17.4	1	3	5	5
1126	2	31	27	4.3	0	2	0	0	1	0	0	0	0.756	−0.28440367	7.9	7.9	1	3	5	5
1128	2	67	64	4.3	0	2	0	1	0	0	0	0	0.54	−2.26605505	14.3	15.5	1	3	5	5
1130	2	36	31	5	0	3	0	0	1	0	0	0	0.884	0.889908257	11.1	8.9	1	0.3	5	0
1132	2	61	59	2	0	2	0	1	0	0	0	0	0.715	−0.66055046	13.8	11.8	1	0.3	5	0
1133	2	30	27	3	0	2	0	0	0	1	0	0	0.733	−0.49541284	14.1	12.5	1	2.8	5	5
1135	2	67	48	19	0	2	0	0	0	1	0	0	0.531	−2.34862385	8.2	9.1	1	10	20	5
1137	2	31	23	8	0	5	0	0	0	0	0	0	0.701	−0.78899083	6.6	11.5	1	7	50	5
1138	2	75	73	2.1	0	2	0	0	0	1	0	0	0.536	−2.30275229	16.4	12.4	1	1.3	5	0
1139	2	82	77	5	0	1	0	0	0	0	1	0	0.528	−2.37614679	10.1	11.8	1	2	20	2.5
1141	2	27	22	5	0	3	0	0	1	0	0	0	0.88	0.853211009	7.3	7.5	1	4.8	10	5
1142	2	74	22	52	0	3	0	0	1	0	0	0	0.49	−2.72477064	15.8	12.6	1	36	50	5
1143	2	50	50	0.6	0	1	0	0	1	0	0	0	0.756	−0.28440367	15.3	9.5	0			
1144	1	63	56	7	0	2	0	0	1	0	0	0	0.865	0.016129032	18.5	14.7	1	6.7	60	5
1146	1	68	65	2.5	1	5	0	0	0	1	0	0	0.84	−0.18548387	11.4	12.3	1	1.7	60	5
1147	1	46	43	3	0	2	0	0	0	1	0	0	0.73	−1.07258065	13.9	10.1	1	0.4	5	5
1148	2	44	42	1.8	0	2	0	0	1	0	0	0	0.724	−0.57798165	8.6	14.5	1	0.8	5	5
1149	2	46	40	6	0	3	0	0	0	1	0	0	0.734	−0.48623853	10.2	12.9	1	1	5	5
1150	1	41	33	8	1	3	0	1	0	0	0	0	0.662	−1.62096774	30.5	7.9	1	7.7	30	10
1151	2	64	59	5	0	2	0	0	1	0	0	0	0.739	−0.44036697	32.7	24.5	1	2	10	0
1152	2	35	27	8	0	2	0	0	0	0	1	0	0.762	−0.2293578	7.2	13	1	0.1	5	5

1153	2	36	31	5	0	4	0	0	0	1	0	0	0.875	0.80733945	9.4	13.1	1	4	15	10
3001	2	64.7	52	12.7	0	2	0	0	1	0	0	0	0.582	−1.88073394	23.6	27.2	1	6	40	0
3002	2	33.9	17	16.9	0	4	0	0	1	0	0	0	0.684	−0.94495413	13.4	13.8	1	12	60	0
3003	2	64.1	50.5	13.6	0	2	0	0	1□	0	0	0	0.521	−2.44036697	11.1	13.6	0			
3006	2	68.8	57.8	11	1	4	0	0	1	0	0	0	0.481	−2.80733945	9.4	9.9	1	11	50	5
3007	1	64.9	57.3	7.6	0	3	1	0	0	0	0	0	0.695	−1.35483871	12	12.8	1	3.9	60	0
3008	2	43.1	23.3	19.8	0	5	0	0	0	1	0	0	0.631	−1.43119266	5.8	8.4	1	12.3	50	7
3009	1	58.2	47.5	10.7	0	4	0	0	0	1	0	0	0.933	0.564516129	18.9	24.1	1	9.8	40	0
3012	1	67	54.9	12.1	0	3	0	0	1	0	0	0	0.778	−0.68548387	16.7	13.6	1	11.5	60	5
3016	1	54.3	41.5	12.8	1	5	0	0	1	0	0	0	0.613	−2.01612903	8.1	10.1	1	12.9	60	10
3017	1	64.3	48.8	15.55	0	3	1	0	0	0	0	0	1.053	1.532258065	10.1	18.8	1	9.2	60	0
3018	2	49.9	34	15.9	0	4	0	0	0	0	1	0	0.717	−0.64220183	5.9	8.6	1	13.7	40	2.5
3019	2	54.3	47	7.25	1	4	0	0	0	1	0	0	0.518	−2.46788991	6.8	11.2	1	6.61	60	5
3020	2	64.9	46	18.9	0	3	0	0	1	0	0	0	0.685	−0.93577982	13.8	12	1	1.1	35	0
3021	2	54.9	49	5.9	1	4	0	0	0	1	0	0	0.523	−2.42201835	7.8	10.2	1	4.58	40	7
3022	2	38.5	28.2	10.3	0	2	1	0	0	0	0	0	0.964	1.623853211	8	10.9	1	3.5	50	0
3023	1	77.7	70.6	7.1	0	2	0	0	1	0	0	0	0.68	−1.47580645	14.6	12.7	1	7	60	5
3024	1	71.8	54.5	17.3	1	5	0	0	0	1	0	0	0.685	−1.43548387	11.3	23.9	1	2.2	60	0
3026	1	74.6	66.8	7.8	0	2	1	0	0	0	0	0	0.648	−1.73387097	12.7	16	1	5.3	60	0
3027	1	76.4	50	26.4	0	1	0	0	0	1	0	0	0.643	−1.77419355	16.4	13.9	1	0.9	15	0
3028	2	64.4	50.9	13.5	1	4	0	0	1	0	0	0	0.761	−0.23853211	7.1	10.6	1	13.5	40	6.25
3029	1	49.3	22	27.25	0	2	0	0	0	1	0	0	0.797	−0.53225806	12.5	11.4	0			
3031	2	32.8	20	12.8	0	3	0	0	0	1	0	0	0.665	−1.11926606	9.4	15.3	1	3.2	10	0
3032	2	76.7	71	5.7	1	2	1	0	0	0	0	0	0.534	−2.32110092	6.9	15.6	1	2.1	30	0
3033	2	42.5	28.1	14.4	0	4	0	0	1	0	0	0	0.706	−0.74311927	4.6	10	1	11.3	60	0
3034	1	56.4	44	12.4	0	1	1	0	0	0	0	0	0.611	−2.03225806	23.7	15.1	1	9.75	40	0
3035	2	87.8	71.9	15.9	0	2	0	0	0	1	0	0	0.548	−2.19266055	9.9	26.8	1	12.3	30	0
3036	1	57.1	51.5	5.6	0	2	0	0	1	0	0	0	0.545	−2.56451613	8.8	14.8	0			
3037	1	70.9	59	11.9	1	3	0	0	0	1	0	0	0.627	−1.90322581	12.4	8.5	0			
3042	2	44.5	38.3	6.2	0	2	0	0	0	1	0	0	0.72	−0.6146789	7.8	8.8	1	3.2	50	0
3043	2	81.1	67.5	13.6	0	4	0	1	0	0	0	0	0.578	−1.91743119	22.3	16.6	1	3.4	60	0
3044	2	68.3	45	23.3	0	3	0	0	0	1	0	0	0.515	−2.49541284	16.5	11.8	1	1	30	0
3045	2	40.5	18.8	21.75	0	2	0	0	0	1	0	0	0.554	−2.13761468	13.2	13.5	1	6.1	40	0
3046	1	37.7	14	23.7	0	2	0	0	0	1	0	0	0.742	−0.97580645	16.1	11.2	1	14.7	50	0
3047	1	84.7	77.8	6.9	0	1	0	0	0	0	1	0	0.602	−2.10483871	10.3	11	0			
3051	2	28.8	18.8	10.05	0	1	1	0	0	0	0	0	0.541	−2.25688073	23	11.4	0			
3052	2	69.9	55.3	14.6	0	2	0	0	0	1	0	0	0.521	−2.44036697	9.4	11	0			
3055	2	71.5	61.7	9.8	1	2	1	0	0	0	0	0	0.574	−1.95412844	4.3	9.2	0			
3057	2	42.3	24.1	18.65	0	2	0	0	0	1	0	0	0.777	−0.09174312	13.4	10.8	0			
3059	1	38.7	34.9	3.8	1	3	0	0	0	1	0	0	0.683	−1.4516129	30.8	12.4	1	2.9	30	7
3060	2	80.2	61.6	18.6	1	3	0	0	0	1	0	0	0.456	−3.03669725	11.7	16.4	1	17.2	30	2
3061	2	41.5	36.2	5.3	0	3	1	0	0	0	0	0	0.553	−2.14678899	8.6	10.9	1	2.66	50	0
3062	1	63.1	58.1	5	0	1	0	0	0	0	1	0	0.727	−1.09677419	10	9.9	0			
3102	1	32.6	25	7.6	0	3	0	0	0	1	0	0	0.743	−0.96774194	7.2	9.3	1	1.1	30	30
3103	1	20.5	4	16.1	0	2	0	0	1	0	0	0	0.969	0.85483871	10.3	11	1	6.1	50	10
3104	1	65	61	4	1	3	0	0	1	0	0	0	0.961	0.790322581	10.7	12.4	1	3.9	40	2.5
3107	2	52.3	48.4	3.9	0	3	0	0	1	0	0	0	0.578	−1.91743119	6	10	1	3.7	45	3
3108	2	38.8	29	9.8	0	3	0	0	0	1	0	0	0.756	−0.28440367	7.7	2.9	0			
3110	2	49.9	42	7.9	0	4	0	0	0	0	0	0	0.921	1.229357798	12.9	10.1	1	5.6	50	0
3111	1	27.5	19.5	8	1	2	0	0	1	0	0	0	1.027	1.322580645	5.7	9.3	1	3.5	60	0
3112	2	32.6	28.1	4.5	0	5	0	0	1	0	0	0	0.914	1.165137615	12.3	12.7	1	3.6	60	3
3114	2	69.9	40	29.9	0	2	0	0	1	0	0	0	1.183	3.633027523	14.5	14.2	0			
3117	1	67.8	64.1	3.7	0	2	0	0	1	0	0	0	0.569	−2.37096774	19.3	11.6	0			
3118	1	78.4	74.8	3.6	0	2	0	0	1	0	0	0	0.586	−2.23387097	9.7	19.2	1	3	60	10
3119	1	77.2	71.4	5.8	0	2	0	0	0	1	0	0	0.556	−2.47580645	4.6	10.9	1	0.16	5	5
3120	2	49.7	40.2	9.5	1	5	0	0	1	0	0	0	0.739	−0.44036697	14.1	22.9	1	3.5	60	0
3125	2	77.6	74.2	3.4	0	2	0	1	0	0	0	0	0.435	−3.2293578	5.9	10.8	1	2.6	10	5
3126	1	65.3	62.8	2.5	0	1	0	0	0	1	0	0	0.737	−1.01612903	10.6	8.5	1	1.2	10	0
3127	2	72.4	69.7	2.7	0	4	0	1	0	0	0	0	0.666	−1.11009174	7.5	13.9	1	2.25	10	5

3129	2	33.8	10	23.8	0	3	0	0	1	0	0	0	0.536	−2.30275229	8.8	8.8	1	8.3	45	5
3130	2	79.3	76.8	2.5	0	1	0	0	1	0	0	0	0.645	−1.30275229	6.6	9.8	1	2.2	10	5
3131	1	56.9	54.4	2.5	0	1	0	0	0	0	0	1	0.753	−0.88709677	7.9	8.8	1	2.1	15	7
3132	2	76.3	73.8	2.5	0	2	0	0	1	0	0	0	0.38	−3.73394495	10.7	9.3	1	2	10	3
3133	1	62.6	60.5	2.1	1	5	0	0	1	0	0	0	0.661	−1.62903226	8.1	7.4	1	2	30	5
3134	1	70.5	66.9	3.6	0	1	0	0	1	0	0	0	0.933	0.564516129	12.3	10	0			
3138	2	28	26	2	0	2	0	0	0	0	0	1	0.689	−0.89908257	9.2	10	0			
3139	1	58.1	31.1	27	0	4	1	0	0	0	0	0	0.79	−0.58870968	11.1	13.2	1	3.8	60	0
3140	2	61.4	59.2	2.2	0	3	0	0	0	1	0	0	0.481	−2.80733945	14.5	10.8	1	1.9	20	10
3143	2	60	55	5	0	1	0	0	0	0	1	0	0.719	−0.62385321	13.9	15.1	0			
3144	2	43.8	42.4	1.4	1	2	0	0	0	1	0	0	0.689	−0.89908257	5.8	10.8	1	1.3	15	10
3145	2	63.4	61.8	1.6	1	2	0	0	0	1	0	0	0.604	−1.67889908	24.9	16	0			
3146	2	43.6	42.3	1.3	1	3	0	0	1	0	0	0	0.828	0.376146789	7.9	7.2	1	1	20	7.5
3147	1	47.8	46.6	1.16	0	2	0	0	1	0	0	0	0.864	0.008064516	18.1	19.3	1	1	10	10
3148	1	41.7	39.1	2.6	0	1	0	0	0	1	0	0	0.876	0.10483871	9.6	9.5	0			
3149	1	62.1	60	2.1	0	1	0	0	1	0	0	0	0.555	−2.48387097	12.5	14.5	0			
3150	1	68.1	672	0.9	0	1	0	0	1	0	0	0	0.818	−0.36290323	9.4	10.2	0			
3151	2	33.4	31.8	1.6	0	2	0	0	1	0	0	0	0.899	1.027522936	9.7	6.3	1	0.83	10	5
3152	1	42.3	41.5	0.8	1	3	0	0	0	1	0	0	0.813	−0.40322581	11.3	13.4	1	0.4	30	15
3153	2	40	39.3	0.7	0	2	0	0	1	0	0	0	0.764	−0.21100917	9.5	7	0			
3155	2	32	30.3	1.7	0	2	0	0	1	0	0	0	0.678	−1	5.1	9.4	1	0.67	50	25
3158	2	54.7	49.7	5	1	2	0	0	0	0	0	1	0.677	−1.00917431	12.7	10.8	0			
3159	2	32.1	30.8	1.3	0	3	0	0	1	0	0	0	0.775	−0.11009174	5.9	13.8	1	0.38	30	17.5
3160	1	74.7	74.5	0.25	0	1	0	0	0	0	1	0	0.82	−0.34677419	11.9	11.9	0			
3161	2	78.3	76.8	1.5	0	2	0	0	1	0	0	0	0.53	−2.35779817	13.4	13.6	0			
3162	2	70.9	53	17.9	0	2	0	0	1	0	0	0	0.591	−1.79816514	6.2	10.3	1	0.67	10	7.5
3163	2	48.1	47.2	0.9	0	1	0	0	1	0	0	0	0.821	0.311926606	8.4	6.8	0			
5002	1	36	25	11.5	0	5	0	0	0	1	0	0	0.678	−1.49193548	4.2	12.1	1	11.4	80	18
5003	2	50	49	0.83	1	4	0	0	0	0	1	0	0.685	−0.93577982	9.7	16.8	1	0	20	20
5004	2	39	31	8.8	0	3	0	0	0	0	0	0	0.669	−1.08256881	5.9	9.2	1	8.2	50	20
5005	2	44	34	9.5	0	1	0	0	1	0	0	0	0.757	−0.27522936	14.3	10.7	0			
5006	2	68	59	9.5	1	3	0	0	1	0	0	0	0.372	−3.80733945	7.6	14	1	7.2	30	2
5008	1	49	43	6	1	5	0	0	0	1	0	0	0.625	−1.91935484	10.4	12.4	1	2.9	70	15
5009	2	59	54	5.1	0	1	0	0	1	0	0	0	0.617	−1.55963303	12.1	13.4	0			
5011	2	54	40	14	1	2	0	0	0	1	0	0	0.694	−0.85321101	5.5	15.5	0			
5012	2	58	46	11.5	1	3	0	0	0	1	0	0	0.67	−1.0733945	7.6	11	1	11	40	10
5013	1	53	50	2.7	0	2	0	0	0	0	1	0	0.846	−0.13709677	6.8	16.1	1	0.25	25	25
5015	1	62	57	5.4	0	3	0	0	0	1	0	0	0.612	−2.02419355	12.9	18.3	1	4.9	25	2.5
5016	2	63	39	24	0	3	0	0	1	0	0	0	0.532	−2.33944954	10.9	14.5	1	19.1	30	2.5
5017	2	64	58	6.2	0	3	0	0	0	1	0	0	0.644	−1.31192661	6.2	16.6	1	6.1	60	30
5018	1	52	41	11.5	1	4	0	0	0	1	0	0	0.655	−1.67741935	9.4	24	1	11.3	40	12.5
5020	2	26	20	6	0	3	0	0	1	0	0	0	0.642	−1.33027523	9.2	11.5	0			
5024	2	67	57	10	0	2	0	0	0	1	0	0	0.653	−1.2293578	25.1	15.9	1	3.5	15	0
5026	2	43	38	5	0	4	0	0	0	1	0	0	0.7	−0.79816514	7.7	14.8	1	1.3	45	0
5027	1	73	58	15	0	1	0	0	1	0	0	0	0.753	−0.88709677	11	12	0			
5028	1	52	51	0.66	0	2	0	0	1	0	0	0	0.61	−2.04032258	11.2	18	1	0.33	30	25
5030	2	73	65	7.7	1	3	0	0	1	0	0	0	0.621	−1.52293578	14.9	16	1	6.8	30	2.5
5031	1	67	53	13.8	1	3	0	0	0	0	1	0	0.556	−2.47580645	12.3	12.2	1	13.2	40	10
5032	1	56	51	5.2	0	2	0	0	1	0	0	0	0.924	0.491935484	10	27.2	1	4.7	50	5
5033	2	60	23	37	0	2	0	0	0	1	0	0	0.5	−2.63302752	11.7	18	1	15.8	25	2.5
5034	2	55	50	5.6	1	5	0	0	0	1	0	0	0.859	0.660550459	6.6	22.4	1	5.1	40	10
5035	2	35	35	0.16	0	1	0	0	0	0	1	0	0.807	0.183486239	7.6	18.3	0			
5036	2	70	69	1.1	1	4	0	0	1	0	0	0	0.48	−2.81651376	29.4	15.4	1	0.17	20	20
5038	2	67	56	11	0	5	0	1	0	0	0	0	0.785	−0.01834862	9.1	11.8	1	6.8	10	2.5
5039	2	56	45	11.1	1	3	0	0	0	1	0	0	0.562	−2.06422018	11.5	19.5	1	10.8	60	5
5040	1	73	62	10.5	0	2	0	0	0	1	0	0	0.591	−2.19354839	12	14.6	1	7.7	60	7
5043	1	28	25	3	0	2	0	0	1	0	0	0	0.746	−0.94354839	9.2	14.4	1	2.7	30	10
5044	2	81	72	8.9	1	1	0	0	0	1	0	0	0.588	−1.82568807	17.6	15.5	0			
5045	2	52	29	23	0	2	0	0	0	0	1	0	0.648	−1.27522936	13.4	29.9	1	6.5	25	1

5046	2	39	31	7.9	0	4	0	0	0	1	0	0	0.645	−1.30275229	7.6	23	1	7.9	50	5
5047	1	71	63	8	0	3	0	0	0	0	0	1	0.5	−2.92741935	21.5	18.1	0			
5049	2	68	64	4	0	1	0	0	1	0	0	0	0.55	−2.17431193	14.9	15.2	0			
5050	2	70	64	5.5	0	3	0	0	1	0	0	0	0.593	−1.77981651	12.3	18.6	1	5	10	2.5
5052	2	28	18	10.5	0	2	0	0	0	1	0	0	0.962	1.605504587	6.4	17.6	1	10.1	30	15
5055	1	45	42	2.7	0	2	0	0	0	0	0	0	0.795	−0.5483871	12	17.9	1	2	20	5
5056	2	50	33	16.9	0	2	0	0	0	1	0	0	0.735	−0.47706422	13.1	15.2	1	10	50	5
5059	2	52	42	10.6	0	3	0	0	1	0	0	0	0.91	1.128440367	13.1	23.6	0			
5060	2	40	27	12.8	0	2	0	0	0	1	0	0	0.549	−2.18348624	6.5	12.4	1	11.5	25	10
5061	2	61	48	13	0	3	0	0	1	0	0	0	0.515	−2.49541284	17.5	18.5	1	12.8	20	2.5
5064	1	71	58	13	1	5	0	0	0	1	0	0	0.892	0.233870968	15.6	17.7	1	12.6	60	2.5
5068	2	64	42	22	0	2	0	0	0	1	0	0	0.47	−2.90825688	29.5	23.7	0			
5069	1	78	67	11.5	0	3	0	0	0	1	0	0	1.072	1.685483871	8.9	12	1	10.9	50	5
5079	1	51	40	11	1	5	0	0	0	1	0	0	0.802	−0.49193548	8.8	14.5	1	12	40	11
6001	1	46	45	1.5	1	3	0	0	0	1	0	0	0.779	−0.67741935	13.6	16.5	1	0.9	20	15
6002	2	40	28	12.5	0	2	0	0	1	0	0	0	0.762	−0.2293578	9.9	17.4	1	4.8	10	9
6009	1	67	63	4	0	1	0	0	0	1	0	0	0.82	−0.34677419	15.9	16.8	1	2.7	30	1
6012	1	70	66	4.5	0	3	0	0	0	1	0	0	0.713	−1.20967742	8.3	11.2	1	3.9	60	15
6013	1	75	71	4.5	0	2	0	0	0	1	0	0	0.922	0.475806452	13.9	14.4	1	4.2	50	5
6014	1	75	68	6.5	0	1	0	0	0	1	0	0	0.873	0.080645161	12	13.9	1	5.7	10	10
6015	2	54	50	3.5	0	2	0	0	0	0	1	0	0.755	−0.29357798	11.1	13.8	1	2.5	45	0
6016	1	80	74	5.8	0	2	0	0	1	0	0	0	0.653	−1.69354839	10.9	14	1	3.4	30	2.5
6017	2	49	46	2.6	0	2	0	0	0	0	1	0	0.777	−0.09174312	8.3	13.5	1	0.3	15	10
6020	1	71	53	18.1	0	1	0	0	1	0	0	0	0.745	−0.9516129	13.3	10.3	1	8.6	15	5
6021	2	30	26	3.7	0	2	0	0	0	1	0	0	0.672	−1.05504587	8.2	14.6	1	1.9	15	10
6028	2	24	15	9	0	2	0	0	0	0	1	0	0.734	−0.48623853	9.6	10.6	1	3.5	20	7.5
6031	1	55	53	2	0	1	0	1	0	0	0	0	0.897	0.274193548	10.4	15	0			
6032	2	39	33	6.6	0	2	0	0	0	0	1	0	0.669	−1.08256881	7.5	10.4	1	5.8	40	5
6035	1	69	57	12.7	0	2	0	0	0	1	0	0	1.011	1.193548387	8.6	15.5	1	1	10	0
6036	2	78	73	5.7	0	2	0	0	0	1	0	0	0.496	−2.66972477	12.2	18.2	1	2	20	0
6041	2	48	37	11.4	0	3	0	0	0	1	0	0	0.698	−0.81651376	7.7	14	1	1.2	15	10
6043	2	46	25	20.7	1	3	0	0	0	1	0	0	0.742	−0.41284404	15.5	15.3	1	15	60	7
7002	2	66	24	42	0	2	0	0	0	1	0	0	0.42	−3.36697248	14	12.1	1	20	40	7.5
7003	2	81	44	45	0	1	0	0	1	0	0	0	0.406	−3.49541284	32	17	1	40	10	10
7004	1	64	38	26	0	2	0	0	1	0	0	0	0.576	−2.31451613	29	14.1	1	2.3	60	0
7005	2	40	19	20	0	4	0	0	0	1	0	0	0.592	−1.78899083	9	19.2	1	20	50	7
7006	2	71	48	23	0	4	0	0	0	1	0	0	0.687	−0.91743119	6	13	1	9	40	0
7007	2	64	22	42	0	5	0	0	1	0	0	0	0.734	−0.48623853	13	35.4	1	26	22.5	0
7008	1	71	50	21	0	2	1	0	0	0	0	0	0.74	−0.99193548	10	12.5	1	2	40	0
7010	2	65	50	15	0	1	0	0	1	0	0	0	0.56	−2.08256881	12	12.1	0			
7011	1	71	55	16	0	3	0	0	1	0	0	0	0.703	−1.29032258	8	8.2	1	16	50	8.5
7012	2	67	35	32	0	3	0	0	0	1	0	0	0.807	−1.65137615	13	13.6	1	1.5	50	0
7014	2	69	5	67	0	1	0	0	0	0	0	0	0.7	−0.79816514	10	14.8	1	11.8	35	2
7015	2	62	10	52	0	2	0	0	0	1	0	0	0.772	−0.13761468	12	12.4	1	1.5	50	0
7016	2	32	17	15	0	2	0	1	0	0	0	0	0.716	−0.65137615	13	12.1	1	14	40	4.5
7017	2	58	27	31	0	3	0	0	0	0	1	0	0.561	−2.0733945	13	14.1	1	0.3	30	0
7018	2	64	36	28	0	1	1	0	0	0	0	0	0.515	−2.49541284	15	10.4	0			
7019	2	78	46	27	1	3	0	0	0	1	0	0	0.561	−2.0733945	22	20.4	1	3	15	0
7020	2	66	53	13	1	4	0	0	0	1	0	0	0.681	−0.97247706	20	12	1	13	50	2
7021	2	66	39	27	0	5	0	0	0	1	0	0	0.459	−3.00917431	9	13.4	1	10	50	3.5
7022	2	62	48	13	0	2	0	1	0	0	0	0	0.795	0.073394495	9	9.4	1	13	50	5.5
7024	2	39	28	11	0	1	1	0	0	0	0	0	0.751	−0.33027523	11	10.2	0			
7025	1	49	39	10	1	2	0	1	0	0	0	0	0.753	−0.88709677	9	11.4	1	10	40	2.5
7027	2	57	49	8	0	1	0	0	1	0	0	0	0.769	−0.16513761	6	10.3	0			
7029	1	55	48	7	1	2	0	0	0	1	0	0	0.817	−0.37096774	14	11.7	1	7	50	2
7030	2	39	30	9	0	2	0	0	0	1	0	0	0.674	−1.03669725	8	3.8	1	2.7	30	10
7031	2	67	61	8	0	2	0	0	0	1	0	0	0.519	−2.4587156	13	10.6	1	6.2	40	2
7032	2	85	79	6	0	2	0	0	1	0	0	0	0.501	−2.62385321	15.5	9.5	1	2	2.5	0
7034	2	65	60	5	0	1	1	0	0	0	0	0	0.722	−1.49541284	21	20.5	0			

7035	2	76	71	5	1	4	0	0	0	1	0	0	0.5	−2.63302752	12	24.4	1	4.8	60	2.5
7036	2	84	80	4	0	5	0	0	0	1	0	0	0.341	−2.01834862	20	27.7	1	3.9	47.5	0
7037	2	60	49	11	0	2	0	0	0	1	0	0	0.519	−2.4587156	6	8.5	1	2.5	20	3.5
7038	2	55	52	3	1	1	0	0	1	0	0	0	0.67	−1.0733945	13	14.7	1	2.8	15	45
7039	2	90	86	4	0	1	0	0	0	1	0	0	0.411	−3.44954128	11	16.8	1	3	15	1.5
7040	2	47	43	4	0	1	0	0	0	1	0	0	0.832	0.412844037	10	12	1	3.5	15	5.5
7041	2	48	44	4	0	2	0	0	0	1	0	0	0.649	−1.26605505	7	8.3	1	4	45	5.5
7042	2	75	72	3	0	1	0	0	1	0	0	0	0.515	−2.49541284	12	14	0			
7043	2	70	67	3	0	3	0	0	0	1	0	0	0.712	−0.68807339	8	10.9	1	2.9	45	2
7044	1	51	50	1.7	0	1	0	0	1	0	0	0	0.818	−0.62096774	19	13.7	0			
7046	1	45	43	2.3	1	3	0	0	0	1	0	0	0.843	−0.16129032	8	12.8	1	21	50	17
7047	1	49	27	22	0	1	0	0	1	0	0	0	0.657	−1.66129032	15	11.7	0			
7048	2	46	44	2.5	0	2	0	0	0	1	0	0	0.681	−0.97247706	6	8.9	1	1.5	50	12.5
7049	2	30	28	2	0	3	0	0	0	1	0	0	0.663	−1.13761468	8.1	6	1	1.9	30	7.5
7050	2	35	33	2	0	1	0	0	1	0	0	0	1.185	2.71559633	7	10.9	0			
7051	2	47	41	6	0	2	0	0	0	0	1	0	0.761	−0.23853211	9.3	29	1	6	20	1.5
7053	2	76	68	8	0	2	0	0	0	1	0	0	0.532	−2.33944954	14	14.7	0			
7054	1	69	67	2	0	1	0	0	0	0	1	0	0.768	−0.76612903	16	12.3	0			
7055	2	62	37	25	0	2	0	0	0	1	0	0	0.586	−1.8440367	10	9.9	1	25	50	25
7056	2	74	48	26	0	5	0	0	0	1	0	0	0.47	−2.90825688	7	8.7	1	26	50	15
7057	1	60	50	10	0	5	0	0	0	1	0	0	0.66	−1.63709677	15	14.4	1	5	50	0
7059	2	63	62	1.2	0	2	0	0	0	0	1	0	0.669	−1.08256881	13	16.3	0			
7060	1	46	40	6	0	2	0	0	0	0	1	0	0.941	0.60483871	16	7.8	1	6	15	9
7063	2	64	60	4	0	1	0	0	0	1	0	0	0.615	−1.57798165	12	13.7	1	4	35	2
7066	2	60	15	45	0	3	0	0	0	1	0	0	1	−0.17431193	10	8.6	1	30	50	2
8001	1	66	48	18	0	1	0	0	1	0	0	0	0.61	−2.04032258	21	13.8	0			
8006	2	71	59	12.3	1	2	0	0	0	1	0	0	0.593	−1.77981651	23	22.7	0			
8007	1	74	67	7	0	5	0	0	0	1	0	0	0.47	−3.16935484	7.4	16.5	1	1.6	10	7
8008	1	82	47	35	0	2	0	0	0	0	1	0	0.71	−1.23387097	9.8	14.9	1	4.5	20	0
8009	2	59	45	15	1	5	0	1	0	0	0	0	0.677	−1.00917431	25.7	22.5	1	5.1	50	0
8010	1	40	36	3.2	1	3	0	0	0	1	0	0	0.645	−1.75806452	6.7	11.8	1	3	30	19
8011	1	61	54	7	0	1	0	0	1	0	0	0	0.699	−1.32258065	9.7	12	0			
8012	2	60	49	10.5	0	1	0	0	0	0	1	0	0.523	−2.42201835	24.6	26	1	10	40	7
8013	2	71	55	15.5	1	3	0	0	0	1	0	0	0.606	−1.66055046	22.5	18.8	1	6.5	27.5	0
8014	1	89	83	6	0	3	0	0	0	1	0	0	0.674	−1.52419355	8	14.1	1	5.9	40	1
8017	2	38	32	6	0	2	0	0	0	1	0	0	0.834	0.431192661	13.6	11.8	0			
8018	2	56	20	36	0	2	0	0	1	0	0	0	0.602	−1.69724771	15.9	13.4	0			
8019	1	83	69	14	0	1	0	0	1	0	0	0	0.549	−2.53225806	10	21.7	1	3.3	7.5	7.5
8020	2	70	66	4.3	0	1	0	0	1	0	0	0	0.599	−1.72477064	22.1	24.6	1	2.1	5	2.5
8021	2	66	56	10	0	2	0	0	1	0	0	0	0.485	−2.7706422	20.7	17.5	1	1	10	6
8022	1	63	55	8	1	3	0	0	0	1	0	0	0.518	−2.78225806	7.3	8.4	1	8	60	13
8023	2	60	57	3.2	1	2	0	0	1	0	0	0	0.653	−1.2293578	12.5	9.2	0			
8024	2	44	43	2	0	1	0	0	0	0	1	0	0.846	0.541284404	8.9	15.5	0			
8025	2	37	30	7.7	0	1	0	0	1	0	0	0	0.811	0.220183486	5.9	8.6	0			
8026	1	65	40	25	0	3	0	0	0	0	1	0	0.551	−2.51612903	17.9	10.3	0			
8027	2	47	25	22.5	1	3	0	0	1	0	0	0	0.687	−0.91743119	7.5	8.3	1	22	40	7.5
8028	2	66	65	1.3	0	1	0	0	1	0	0	0	0.733	−0.49541284	40.3	21.6	0			
8029	2	56	55	1	1	2	0	0	1	0	0	0	0.925	1.266055046	17.9	13.1	0			
8030	2	60	58	2	0	2	0	0	0	0	1	0	0.658	−1.18348624	22.7	18.6	1	0.4	25	0
8032	2	60	49	10.5	0	2	0	0	1	0	0	0	0.551	−2.16513761	16	21.8	1	2	50	0
8037	1	76	59	17.3	1	1	0	0	1	0	0	0	0.689	−1.40322581	17.8	10.8	0			
8038	2	72	57	15	1	2	0	0	1	0	0	0	0.505	−2.58715596	18.9	11.7	1	14.5	35	5
8039	2	69	61	8.3	0	2	0	1	0	0	0	0	0.571	−1.98165138	3.1	10.9	1	4.3	7.5	2.5
8040	2	68	65	3	0	1	1	0	0	0	0	0	0.638	−1.36697248	16.8	15.3	1	0.1	30	0
8042	1	40	37	3	1	2	0	0	1	0	0	0	0.834	−0.23387097	8.7	9.3	1	0.8	30	9
8044	2	54	46	8	0	2	0	0	0	1	0	0	0.656	−1.20183486	18.1	15.7	0			
8045	2	58	51	7.3	1	5	0	0	1	0	0	0	0.558	−2.10091743	11.5	11.5	1	2	50	10
8047	2	43	30	13	0	2	0	0	0	1	0	0	0.74	−0.43119266	5.7	10.3	1	12	50	10
8048	1	62	42	20	1	5	0	1	0	0	0	0	0.733	−1.0483871	8.7	8.3	1	18.7	60	12

8049	2	59	46	12.7	0	2	0	0	0	0	1	0	0.752	−0.32110092	5.8	9	1	10.5	30	11
8050	2	49	32	17	0	2	1	0	0	0	0	0	0.656	−1.20183486	15.8	21.3	0			
8052	2	73	29	44	0	2	0	1	0	0	0	0	0.497	−2.66055046	10.1	11.6	1	4.2	40	5
8054	1	76	73	2.7	0	3	0	0	0	1	0	0	0.809	−0.43548387	7.6	10.5	1	2.1	30	15
8055	2	33	25	8	0	2	0	0	0	1	0	0	0.67	−1.0733945	7.4	9.2	1	1.8	10	6
8056	1	45	43	1.7	0	1	0	0	1	0	0	0	0.796	−0.54032258	16.7	10.8	0			
8057	1	74	72	1.7	0	5	0	0	0	1	0	0	0.736	−1.02419355	5.8	11.9	1	1.5	10	6
8060	1	68	61	7	1	2	0	0	0	0	1	0	0.854	−0.07258065	8.3	13.3	1	6.2	30	10
8061	2	54	54	0.9	1	2	0	0	0	1	0	0	0.613	−1.59633028	17.5	15.3	1	0.3	10	0
8062	1	65	60	4.8	0	4	0	0	0	1	0	0	0.749	−0.91935484	8.1	23.1	1	4	60	25
8063	2	51	20	32	0	5	0	0	0	1	0	0	0.355	−3.96330275	11.3	20.6	1	29	60	24
8065	2	55	24	31	0	2	0	0	1	0	0	0	0.564	−2.04587156	21	14.6	0			

Abbreviations in Table

Onset: onset age of MG, Duration: disease duration of MG, MGFA: clinical severity determined by the recommendations of the Myasthenia Gravis
CSR, PR, MM, I, U, W: post interventional status, BMD: bone mineral density at the femoral neck was measured using a dual-energy X-ray absorp
NTx: pyridinoline cross-linked amino-terminal telopeptide of type I collagen, BAP: bone isoform of alkaline phosphatase, PSL: prednisolone, treatr
QMG: quantitative MG score, MG com: MG composite score, QOL: MG-QOL15 score, BMI: body mass index Bis: bisphosphonates, Vit D: vitamin

PSL(1 yr total)	CNIs	QMG	MG com	QOL 1	BMI	Bis	Vit D	Ca	Fracture	Parents	smoking	PSL 3mo	RA	secondary	Alco	FRAX
912.5	0	8	3	10	23.3	1	0	0	0	0	0	1	0	0	0	1.4
1825	1	21	20	35	19.9	1	0	0	1	0	0	1	0	0	0	46
1825	0	13	7	4	25.1	1	0	0	0	0	0	1	0	0	0	72
	0	3	0	14	22.6				0	0	0	0	0	0	0	14
3650	0	5	3	13	26.1	1	0	0	0	0	0	0	0	0	0	51
	0	16	11	22	26.9	1	0	0	0	0	0	0	0	0	0	22
	0	15	7	21	18.3				0	0	0	0	1	0	0	11
3600	0	9	10	21	21.8	1	0	0	0	0	0	1	0	0	0	48
1825	1	16	12	15	21.9	1	0	0	0	0	0	1	0	0	0	12
2500	1	9	5	20	14.9	1	0	0	1	0	0	1	0	0	0	28
912.5	1	15	9	16	20.3	1	0	0	0	0	0	1	0	0	0	38
2700	0	11	8	14	19.6	0	1	0	1	0	0	1	0	0	0	2
3102.5	1	12	8	27	21.2	1	0	0	1	0	1	1	0	0	0	59
	0	13	11	16	23.1				0	0	0	0	0	0	0	16
0	0	1	0	0	20.8				0	0	0	1	0	0	0	9.8
2555	1	10	5	13	22.9	1	0	0	0	0	0	1	0	0	0	11
	0	1	0	0	24.9				0	0	0	0	0	0	0	60
	0	10	5	5	15.1				0	0	0	0	0	0	0	3.1
912.5	0	2	0	0	19.8				0	0	0	1	0	0	0	5.6
912.5	1	2	0	0	22.8	0	0	0	0	0	1	1	0	0	0	67
912.5	0	6	1	8	28.9	1	0	0	0	0	0	1	0	0	0	0.9
2848	0	13	6	9	20.1	1	0	0	0	0	0	1	0	0	0	0.7
5840	1	11	8	9	26.7	1	0	0	0	0	0	1	0	0	0	11
0	1	7	7	9	23.8	1	0	0	1	0	0	1	0	0	0	46
2555	0	13	11	27	26.7	1	0	0	0	0	0	1	0	0	0	59
1825	1	7	8	26	22.4	1	0	0	0	0	0	1	0	0	0	9.4
	0	19	15	19	20.8				0	0	0	0	0	0	0	0.4
2628	0	5	2	4	21.8	1	0	0	0	0	0	1	0	0	0	63
0	1	6	0	4	26.4				0	0	0	0	0	1	0	53
2920	1	14	12	9	18.5	1	0	0	0	0	0	1	0	0	0	21
	0	3	3	14	23.8				0	0	0	0	0	0	0	75
3650	1	1	0	8	22.9	1	0	0	0	0	0	1	0	0	0	2.1
5475	0	5	4	12	22.8	1	0	0	0	0	0	1	0	0	0	53
3650	0	9	5	23	22.2	0	1	0	0	0	0	1	0	0	0	0.7
5475	1	12	8	9	24.2	0	1	0	0	0	0	1	0	0	0	0.7
	0	4	3	24	19.5				0	0	0	0	0	0	0	5
4380	1	10	12	40	21.6	1	0	0	0	0	0	1	0	0	0	4.8
1825	1	2	0	5	19.4	1	0	0	0	0	0	0	0	1	0	63
	0	3	1	0	26.4				0	0	0	0	0	0	0	77
2730	0	2	0	13	17.6	0	1	0	0	0	0	1	0	0	0	0.8
	0	14	10	27	21.1	0	0	0	0	0	0	0	0	0	0	0.9
3650	0	8	2	23	19.1	0	1	0	0	0	0	1	0	0	0	0.7
2190	0	9	6	10	22.7	1	0	0	0	0	0	1	0	0	0	64
	0	3	1	21	21.9				0	0	0	0	0	0	0	4.1
2475	1	13	12	31	16.7	1	0	0	0	0	0	1	0	0	0	30
0	1	11	5	1	19				0	0	0	1	0	0	0	1.8
2555	1	4	1	7	20.7	1	0	0	0	0	0	1	0	0	0	9.7
2190	1	9	5	18	26.4	1	0	0	0	0	0	0	0	0	0	7.5
2628	0	7	4	6	25.1	1	0	0	0	0	0	1	0	0	0	56
	0	7	2	11	21.4				0	0	0	0	0	0	0	16
2920	1	2	0	1	20.9	1	0	0	0	0	0	1	0	0	0	2.9
4560	1	4	2	9	22.8	1	0	0	0	0	0	1	0	0	0	1
4800	0	10	18	15	19.1	1	0	0	0	0	0	1	0	0	0	0.7
3650	1	13	12	24	19.1	1	0	0	0	0	0	1	0	0	0	0.7
4015	1	10	9	35	21.6	1	0	0	0	0	0	1	0	0	0	5.2
1752	1	10	8	17	21.6	1	0	0	0	0	0	1	0	0	0	0.7

4500	1	14	10	10	19.7	1	0	0	0	0	0	1	0	0	0	0.7
1620	0	2	0	4	19.4	0	1	0	0	0	0	0	0	0	0	0.4
912.5	1	7	4	1	26.5	1	0	1	0	0	0	1	0	0	0	18
1825	1	3	3	6	26	1	0	0	0	0	0	1	0	0	0	8.2
2196	1	15	16	53	21.8	1	0	0	0	0	0	1	1	0	0	0.8
1825	1	1	1	0	25	1	0	0	0	0	1	1	0	0	1	41
913	1	1	1	2	21.9	1	0	0	0	0	0	1	0	0	0	0.7
1825	1	3	0	3	26				0	0	1	1	0	0	1	78
1825	1	1	0	5	20.4	0	1	0	0	0	0	1	0	0	0	0.7
0	1	7	5	17	25.1	0	1	0	0	0	0	1	0	0	0	3.3
1825	1	13	11	29	20.9	1	0	0	0	0	0	1	0	0	0	6.5
1825	1	8	5	8	20.2	1	0	0	0	0	0	1	0	0	0	9.8
1825	1	4	1	9	24.2	1	0	0	0	0	0	1	0	0	0	14
2738	1	3	2	34	22	1	0	0	0	0	1	1	0	0	0	30
1825	1	7	5	17	24.4	1	0	0	0	0	0	1	0	0	0	7.7
1825	1	2	0	0	17.9	0	1	0	0	0	0	1	0	0	0	0.7
1825	1	3	3	16	24.4				0	0	0	1	0	0	0	65
0	1	5	3	5	28	0	0	1	0	0	0	1	0	0	0	2.2
913	1	5	1	4	26	1	0	0	0	0	0	1	0	0	0	7.9
0	1	0	0	0	28.7				0	0	0	1	0	0	0	8.2
3650	1	6	5	19	19.5	1	0	0	0	0	0	1	0	0	0	0.7
1825	0	1	0	2	21.9	0	1	0	0	0	0	1	0	0	0	0.7
1825	0	0	0	4	28	1	0	0	0	0	0	1	0	0	1	63
1825	1	4	1	4	24.1				0	0	0	1	0	0	0	12
0	1	4	3	13	22.7				0	0	0	1	0	0	1	52
2738	1	13	8	27	22.8	1	0	0	0	0	0	1	0	0	0	5.5
913	1	4	0	8	23.9	0	1	0	0	0	0	1	0	0	0	0.7
1825	0	14	17	37	15.4				0	0	0	1	0	0	0	21
0	1	0	0	0	20.5				0	0	0	1	0	0	0	0.7
0	0	5	2	5	25.3				0	0	0	1	0	0	0	17
0	1	4	0	0	22.3	1	0	0	0	0	0	1	0	0	0	27
2738	0	0	0	0	18.8	0	1	0	0	0	0	1	0	0	0	16
1825	1	10	7	28	31.7	1	0	0	0	0	0	1	0	0	0	12
3650	1	6	5	38	29.1	1	0	0	0	0	0	1	0	0	0	6.5
0	1	11	13	9	20.7				0	0	0	1	0	0	1	28
1825	1	7	5	12	20	1	0	0	1	0	0	1	0	0	0	52
0	1	4	0	5	18.5				0	0	0	1	0	0	0	3.4
	1	7	2	11	25				0	0	0	0	0	0	0	13
1825	1	3	2	23	23.1				1	0	0	1	0	0	0	24
1825	1	7	4	1	17	0	1	0	0	0	0	1	0	0	0	0.7
1825	1	2	0	3	20.9	0	1	0	0	0	0	1	0	0	0	15
0	1	7	6	6	23.9				0	0	1	1	0	1	0	1
0	1	4	4	9	20.5	0	1	0	0	0	1	1	0	0	0	11
1825	1	9	5	21	20	0	1	0	0	0	0	1	0	0	0	0.7
1825	1	7	5	4	18.3	1	0	0	0	0	0	1	0	0	0	16
3200	1	20	33	51	25.2	0	1	0	0	0	0	1	0	0	0	0.6
1050	1	7	1	17	22.2				0	0	0	1	0	0	0	25
913	0	9	5	6	25.3	1	0	0	1	0	0	1	0	0	0	49
1825	1	7	5	28	22.1	0	1	0	0	0	0	1	0	0	0	0.7
2715	1	10	9	13	18.8	1	0	0	1	0	0	1	0	0	0	42
	0	2	1	6	22.3				0	0	0	0	0	0	0	2.9
1825	1	5	5	6	25.2				0	0	0	1	0	1	1	63
3194	1	9	11	37	21.5				0	0	1	1	0	0	1	68
1825	1	9	9	33	25.7				0	0	1	1	0	0	1	46
1460	1	3	2	15	18.9	0	1	0	0	0	0	1	0	0	0	2.2
1825	1	7	9	32	22.5	0	1	0	0	0	0	1	0	0	0	2.9
3650	1	1	0	4	20.1	0	1	0	0	0	1	1	0	1	1	41
0	1	7	2	8	24.3	0	0	1	0	0	0	1	0	0	0	11
	1	14	11	19	20.7	0	1	0	0	0	0	1	0	0	0	0.7

4015	1	13	7	35	29.8	1	0	0	1	0	1	1	0	0	0	3.2
0	1	3	1	2	20.8	0	1	1	0	0	0	0	0	0	0	7.7
0	0	7	2	2	31	0	1	0	0	0	0	0	0	0	0	0.3
	0	7	1	4	18.9				0	0	0	0	0	0	1	9.3
2287	1	7	7	13	17.9	1	0	0	0	1	0	0	0	0	0	18
0	0	3	0	0	26.1	0	1	1	0	0	1	1	0	0	0	64.9
3100	1	7	2	3	18.7	1	1	0	0	0	0	1	0	0	0	1.8
150	1	9	6	6	30.5	0	1	0	0	0	0	1	0	0	0	58.2
4117	1	0	0	11	22.4	1	0	0	0	0	1	1	0	0	0	67
3202	1	2	4	2	21.3	1	1	0	0	0	0	1	0	0	0	54.3
0	1	3	0	22	23.5				0	0	1	1	0	0	0	64.3
912.5	1	22	24	22	20.4	1	1	1	0	0	0	1	0	0	0	4.8
1830	1	5	5	10	16.2	1	1	0	0	0	0	1	0	0	0	6.8
0	0	5	4	7	25.7	0	1	1	0	0	0	1	0	0	0	11
4065	1	11	3	18	22.7	1	1	1	0	0	0	1	0	0	0	6.7
0	0	0	0	0	21.9				0	0	1	1	0	0	0	0.8
1830	0	2	0	0	24.2				0	0	0	1	0	0	0	77.7
0	1	3	2	7	20.3	0	1	1	0	0	0	1	0	0	0	71.8
0	0	2	0	0	22.5				0	0	0	0	0	0	0	74.6
0	0	7	6	5	29.1				0	0	0	1	0	0	0	76.4
2356	1	4	1	3	27.2	1	1	0	0	0	0	1	0	0	0	10
	0	6	6	4	26.9				0	0	0	0	0	0	0	49.3
0	0	11	7	9	19.1				0	0	0	1	0	0	0	0.7
0	0	2	0	12	26.4	1	1	0	0	0	0	1	0	0	0	26
0	1	5	0	4	20.2	0	1	1	0	0	1	1	0	0	1	2.3
0	0	1	0	0	22.5				0	0	0	1	0	0	0	56.4
0	1	12	3	3	24.3	1	1	0	0	0	0	1	0	0	0	32
	0	5	1	7	28.8				0	0	0	0	0	0	0	57.1
	1	12	6	16	21.1				0	0	0	0	0	0	1	70.9
0	0	10	3	5	18.6	1	0	0	0	1	0	1	0	0	0	4.9
0	1	3	0	4	20.6	0	1	0	0	0	0	1	0	0	0	36
0	0	6	6	25	21.9				0	0	0	1	0	0	0	15
0	1	13	12	32	19.6				0	0	0	1	0	0	0	1.1
0	0	6	4	2	23.2				0	0	0	0	0	0	0	37.7
	0	6	4	35	23.6				0	0	0	0	0	0	0	84.7
	0	3	0	1	18.5				0	0	0	0	0	0	0	0.4
	0	5	7	6	18				1	1	0	0	0	0	0	34
	0	4	0	6	21.1				0	0	0	0	0	0	0	13
	0	8	5	14	28.7				0	0	0	0	0	0	0	0.7
4020	1	6	8	20	30.2	1	1	0	0	0	0	1	0	0	0	38.7
641	1	6	2	44	18.6	1	0	0	0	0	0	1	0	0	0	37
0	0	2	0	3	17.5				0	0	0	0	0	0	0	0.9
	0	8	7	31	26.6				0	0	0	0	0	0	1	63.1
6990	0	12	4	3	21.4	1	1	0	0	0	0	1	0	0	0	32.6
3955	0	1	0	8	19.3	1	0	0	0	0	0	1	0	0	0	20.5
1189.5	0	2	0	13	21.4	1	0	0	0	0	0	1	0	0	0	65
1460	0	7	3	6	19.2	1	1	0	0	1	0	1	0	0	0	11
	1	11	4	22	25.1				0	0	1	0	0	0	0	0.4
0	0	2	1	7	28	0	1	0	0	0	0	1	0	0	0	4.3
0	1	2	0	0	23.6	1	1	0	0	0	0	1	0	0	0	27.5
980	0	8	1	5	19.9	0	1	0	0	0	0	1	0	0	0	0.7
	0	3	0	25	30.4				0	0	0	0	0	0	0	8.5
	0	3	0	1	23.2				0	0	0	0	0	0	0	67.8
5490	1	4	0	19	22.4	0	1	0	0	0	0	0	0	0	0	78.4
300	0	5	1	19	19.8	1	0	0	0	0	0	1	0	0	0	77.2
0	0	1	1	3	24.5				0	0	0	1	0	0	0	4.7
2007	0	2	0	3	29	1	0	0	0	0	0	1	0	0	0	26
2100	0	3	4	21	25.8	1	0	0	0	0	0	1	0	0	0	65.3
1825	1	2	0	11	28	1	0	0	0	0	0	1	0	0	0	17

1825	0	4	0	2	18.9	1	1	0	0	0	0	1	0	0	0	0.7
1830	0	4	0	3	30.6	1	0	0	0	0	0	1	0	0	0	26
3111	0	8	7	24	24.1	1	1	0	0	0	1	1	0	0	0	56.9
1098	0	4	1	15	22.6	1	0	0	0	0	1	1	0	0	0	30
1890	1	2	0	0	24.5	1	0	0	0	0	0	1	0	0	0	62.6
	0	0	0	1	27.7				0	0	1	0	0	0	0	70.5
	0	12	13	20	23.1				0	0	0	0	0	0	0	0.4
0	0	1	0	0	26				0	0	0	1	0	0	0	58.1
5490	1	7	3	39	22.4	1	1	0	0	0	0	1	0	0	0	9.4
	0	9	7	16	20.6				0	0	0	0	0	0	0	5.5
4110	1	3	4	7	18.9	0	1	0	0	0	0	1	0	0	0	2.2
	0	7	4	20	21.4				0	0	0	0	0	0	0	6.7
3782.5	0	3	1	1	23.4	0	1	0	0	0	0	0	0	0	0	1.3
3650	0	0	0	5	21.2				0	0	1	1	0	0	0	47.8
	0	7	3	2	22.9				0	0	0	0	0	0	0	41.7
	0	6	1	0	17.6				0	0	1	0	0	0	1	62.1
	0	1	0	1	26				0	0	0	0	0	0	0	68.1
2446	0	3	0	2	27.2	0	1	1	0	0	0	0	0	0	0	0.4
2195	0	8	6	19	33.6				0	0	0	1	0	0	0	42.3
	0	7	0	9	22.2				0	0	0	0	0	0	0	0.4
6405	0	3	0	8	22	1	0	0	0	0	0	1	0	0	0	0.7
	0	7	4	42	23.9				0	0	0	0	0	0	0	4.1
2995	0	2	0	5	18	0	1	0	0	0	0	1	0	0	0	0.7
	0	13	7	20	24.9				0	0	0	0	1	0	0	74.7
	0	4	0	0	27.4	0	1	0	0	0	0	0	0	0	0	13
2145	0	2	4	1	20.8	1	0	0	0	0	0	1	0	0	0	19
	0	2	1	4	21.6				0	0	1	0	0	0	0	2.5
8319	1	17	23	17	32	1	0	0	1	0	0	1	0	0	0	36
0	0	19	21	19	26				0	0	0	0	0	0	0	2.8
5255	1	13	10	44	18.1	1	1	0	0	0	0	1	0	0	0	0.7
	0	4	5	48	28.5				0	1	0	0	0	0	0	2.3
730	1	9	9	17	20.4	1	0	1	0	0	0	1	0	0	0	16
5475	1	14	11	16	27.1	1	0	0	1	0	1	1	0	0	0	49
	0	6	6	5	19.8				0	0	0	0	0	0	0	31
	0	18	22	19	18.6				0	0	0	0	0	0	0	4
3650	1	19	24	33	29.6	1	1	0	0	0	0	1	0	0	0	6.8
1725	0	7	7	30	25	0	1	0	1	0	0	1	0	0	1	53
912.5	1	10	13	8	31.6	0	0	1	0	0	0	1	0	0	0	62
912.5	0	8	6	3	18.7	0	1	0	0	0	0	1	0	0	0	12
5785	1	7	10	44	27.3	1	0	0	0	0	0	1	0	0	0	10
4562	1	12	19	38	27.1	1	0	1	0	0	0	1	0	0	0	52
	0	3	3	0	19.3				0	0	0	0	0	0	0	0.4
0	0	18	22	3	17.3				0	0	0	1	0	1	0	23
337.5	1	18	23	31	19.1	1	0	0	0	0	0	1	0	0	0	0.6
	0	5	2	4	27.5				0	0	0	0	0	0	0	73
1885	1	6	1	3	27.4	1	0	0	0	0	0	1	0	0	0	52
1237.5	1	8	11	0	21.8	0	0	1	0	0	0	1	0	0	0	22
3825	1	16	17	28	20	0	1	0	0	0	1	1	0	0	0	67
1825	0	3	2	6	31.3	1	0	0	0	0	0	1	0	0	0	56
912.5	0	14	22	17	23.6				0	0	0	1	0	0	0	8.6
3975	1	9	16	22	23.4	1	1	0	0	0	0	1	0	0	0	6.6
	0	7	4	34	22.5				0	0	0	0	0	0	0	0.4
705	0	4	3	23	24.5	1	0	0	0	1	0	1	0	0	0	25
912.5	0	1	0	0	23.8	1	0	0	0	0	0	1	0	0	0	14
1825	1	19	26	24	20	0	1	0	0	0	0	1	0	0	0	7.3
2670	0	10	16	36	24.5	0	1	1	0	1	0	1	0	0	0	73
4050	1	4	5	13	19.4	1	0	1	0	0	1	1	0	0	0	28
	0	16	18	26	22.4				0	0	0	0	0	0	0	23
365	1	23	31	40	21.2				0	0	0	1	0	0	0	5.4

1825	1	16	18	28	20.3	1	0	1	0	0	0	1	0	0	0	0.7
	1	22	31	37	19				0	0	1	0	0	1	0	71
	0	3	0	11	22.1	1	1	0	0	0	0	0	1	0	0	13
912.5	1	6	5	4	22.5	0	0	1	0	0	0	1	0	0	0	17
6265	1	13	13	6	36.2				0	0	1	1	0	0	0	0.5
1825	0	7	4	23	25.1	1	0	0	0	0	1	1	0	0	0	45
1825	1	11	10	20	34.7	0	1	0	0	0	1	1	0	0	0	3.8
	0	9	10	17	28.4				0	0	0	0	0	1	0	4.1
5480	1	12	9	42	18.7	0	1	0	0	0	0	1	0	0	0	0.7
912.5	1	5	4	0	20.3	0	1	0	0	0	0	1	0	0	0	1.1
912.5	0	14	12	10	28.7	0	1	0	1	0	0	1	0	1	0	71
	0	15	14	13	20.8				0	0	0	0	0	0	0	7.2
1825	0	12	10	6	22.3				0	0	1	1	0	0	0	78
4680	1	18	23	37	24	0	1	0	0	0	0	1	0	0	0	51
4920	1	7	7	11	25.6				0	0	1	1	0	0	0	46
3650	1	3	4	1	22.3				0	0	0	1	0	0	0	49
365	1	3	1	4	26				0	0	0	1	0	0	0	67
4777.5	0	5	3	16	26.4	1	0	1	0	0	0	1	0	0	0	70
1272.5	1	4	2	2	24.5				0	0	0	1	0	0	0	75
435	1	3	1	1	22	0	1	0	0	0	0	0	0	0	0	75
542.5	1	10	9	8	20.4	1	0	0	0	0	0	1	0	0	0	6.4
912.5	1	2	0	4	24				0	0	0	1	0	0	0	80
1375	1	10	10	25	27.9				0	0	0	1	0	0	0	3.9
1825	1	1	0	0	25.8				0	0	0	1	0	0	0	71
3650	1	3	1	19	19.8	1	0	0	0	0	0	1	0	0	0	0.7
3697.5	1	8	8	14	21.1	0	1	0	0	0	1	1	0	0	0	0.8
	1	2	0	4	20.4				0	0	0	0	0	0	0	55
1825	0	7	7	20	18	1	1	0	0	0	0	1	0	0	0	0.7
0	1	5	4	1	21				0	0	1	1	0	0	0	69
0	1	5	3	1	19				0	0	0	1	0	0	0	33
3650	1	5	3	3	23.9				0	0	0	1	1	0	0	5.1
7025.8	1	11	12	30	26.7	1	0	0	0	0	0	1	0	0	0	9.2
2974	1	6	4	25	21.1	1	0	0	1	0	0	1	0	0	0	26
3965	0	2	0	14	20.5	1	0	1	0	0	0	0	0	0	0	24
0	0	0	0	15	21.3				0	0	0	1	0	0	0	64
2839	1	11	9	38	19.5	1	0	0	0	0	0	1	0	0	0	0.7
0	0	6	6	30	30.7	1	0	0	0	0	0	1	1	0	0	20
209	1	3	1	1	24.7	1	0	0	0	0	0	1	0	0	0	11
0	0	2	0	1	26.4				0	0	0	1	0	0	0	71
	0	1	0	16	25.4	1	0	0	0	0	0	0	0	0	0	7.2
2999	1	3	0	6	23.4	1	0	0	0	1	0	1	0	0	0	71
0	1	12	10	36	23.4				0	0	0	1	0	0	0	14
895	1	8	7	36	22.3	1	0	0	0	0	0	1	0	0	0	2.4
0	0	3	1	17	19.5				0	0	1	1	0	0	0	12
2258	1	2	0	6	19.7				0	0	0	1	0	0	0	0.7
0	0	11	7	16	17.8				0	0	0	0	0	0	0	5.1
	0	0	0	0	20.4				0	1	0	0	0	0	0	14
0	0	10	8	15	19.6	1	0	0	0	0	0	0	0	0	0	21
916	1	4	2	33	29.2	0	0	1	0	0	0	1	0	0	0	11
1511	1	6	4	13	20.8	1	0	0	0	1	0	1	0	0	0	23
2510	0	2	0	2	24.7	1	0	0	0	0	0	1	0	0	0	9.8
	0	0	0	0	24.2				0	0	0	0	0	0	0	0.4
1007	0	2	0	0	21.3				0	0	0	1	0	0	0	49
	0	1	0	2	20.9				0	0	0	0	0	0	0	4.6
732	1	4	2	22	20.9	0	1	0	0	0	0	1	0	0	0	55
4749	0	6	5	22	18.1	1	0	0	0	0	0	1	0	0	0	0.7
763	0	8	8	14	16.8	1	0	0	0	0	0	1	0	0	0	16
49	1	2	0	0	19	1	0	0	0	0	0	1	0	0	0	38
	0	2	0	0	20.8				0	0	0	0	0	0	0	7.7

992	1	18	16	39	21.5	1	0	0	0	0	0	1	0	0	0	27
92	1	5	4	24	21.5	1	0	0	0	0	0	1	0	0	0	36
1216	0	3	1	14	17.1	1	0	0	0	0	0	1	0	0	0	9.6
3662	0	2	0	15	23.4	1	0	0	0	0	0	1	0	0	0	6.6
697	1	4	2	11	20.1	1	0	0	0	0	0	1	0	0	0	32
2213	0	3	1	19	28.9	0	1	0	0	0	0	1	0	0	0	3
4960	1	3	3	5	32	1	0	0	0	0	0	1	0	0	0	3.1
	0	2	0	3	22.2				0	0	0	0	0	0	0	10
1160	1	5	5	24	23.8	1	0	0	0	0	0	1	0	0	0	16
	0	1	0	2	23.3				0	0	0	0	0	0	0	51
8332	0	4	3	2	21.7	1	0	0	0	0	0	1	0	0	0	45
	1	0	0	7	19.9				0	0	0	0	0	0	0	49
6867	0	5	3	21	20.7	1	0	0	0	0	0	1	0	0	0	2.9
1776	1	6	4	27	17.6	1	0	0	0	0	0	1	0	0	0	0.8
	0	2	0	2	20				0	0	0	0	0	0	0	0.4
549	1	6	4	40	21.8	1	0	0	0	0	0	1	0	0	0	3.4
	1	4	2	30	17.6	1	0	0	0	0	0	0	1	0	0	29
	0	5	3	40	23.4				0	0	0	0	0	0	0	69
916	0	7	5	2	25.2	1	0	0	0	0	0	1	0	0	0	9.4
5494	1	9	7	3	20.5	1	0	0	0	0	0	1	0	0	0	25
0	1	4	2	8	24.9	1	0	0	0	0	1	1	0	0	0	60
	0	5	3	8	21.8				0	0	0	0	0	0	0	0.8
1145	0	9	6	13	28.4	1	0	0	0	0	1	1	0	0	0	46
981	1	3	1	36	22.4	1	0	0	0	0	0	1	0	0	0	12
732	0	7	5	11	22.5	1	0	0	0	0	0	1	0	0	0	8.7
	0	5	4	1	25.6				0	0	0	0	0	0	0	66
	1	12	2	23	26.2				0	1	0	0	0	0	0	16
2820	1	10	10	21	17.7	1	0	0	0	0	0	1	0	0	0	74
0	0	19	11	9	18.4				0	0	0	1	0	0	0	82
0	1	5	0	5	18.9				0	0	0	1	0	0	0	8.8
6400	1	12	8	36	19.8	1	0	0	0	0	1	1	0	0	0	40
	0	2	1	2	23.2				0	0	0	0	0	0	0	61
4000	1	8	5	46	18.9	0	1	0	1	0	0	1	0	0	0	19
0	1	11	6	7	24.4	0	1	0	0	0	0	1	0	0	0	18
1300	0	10	9	44	16.9	1	0	0	0	0	0	1	0	0	0	89
	1	10	11	13	26				0	0	0	0	0	0	0	0.4
	1	7	3	25	24.3				0	0	0	0	0	0	0	4.3
2740	1	9	2	12	20.9				0	0	0	1	0	0	0	83
910	0	4	1	0	21.1				0	0	0	0	0	0	0	11
2700	1	3	1	22	19.6				0	0	0	1	0	0	0	14
5300	1	9	10	26	21.3	1	0	0	0	0	1	1	0	0	0	63
	1	7	8	2	20.4				0	0	1	0	0	0	0	5.9
	1	11	5	24	22.5				0	0	1	0	0	0	0	1.3
	1	9	1	2	26				0	0	1	0	0	0	0	0.4
	0	14	11	30	24.5				0	0	0	0	0	0	0	65
2740	0	7	4	20	20				0	0	0	1	0	0	0	3.4
	0	6	1	15	30.8				0	0	0	0	0	0	0	6.6
	0	4	4	48	17.7				0	1	0	0	0	0	0	8.8
0	1	9	7	29	18				0	0	1	1	0	0	0	10
0	1	7	2	7	19.2				1	0	0	1	0	0	0	19
	1	9	4	5	23.1				0	0	0	0	0	0	0	76
1825	0	6	1	5	24.3				0	0	0	1	0	0	0	19
910	0	6	3	1	28	1	0	0	0	0	0	1	0	0	0	14
440	0	3	0	24	30.4				0	0	0	0	0	0	0	7.6
11385	1	6	1	17	33.8	1	0	0	0	0	1	1	0	0	0	40
	0	11	5	15	23.4				0	0	0	0	0	0	0	3.8
3650	1	5	0	7	23.9	1	1	0	0	0	0	1	0	0	0	7.8
3650	1	13	4	37	26.7	0	0	1	0	0	0	1	0	0	0	1.6
4380	0	4	0	3	23.4				0	0	0	1	0	0	0	62

5040	1	11	14	41	24.3	0	1	0	0	0	0	1	0	0	0	8.1
	0	5	0	8	22.5				0	0	0	0	0	0	0	2.6
2950	0	6	0	2	21.5	0	0	1	0	0	0	1	0	0	0	22
5400	1	12	6	10	27.5	1	0	0	0	0	0	1	0	0	0	76
2966	1	14	13	18	19				0	0	0	1	0	0	0	0.7
	0	2	1	20	32.7				0	0	1	0	0	0	0	45
2850	1	22	16	43	29.7				0	0	0	1	0	0	0	74
3650	1	13	11	33	23.4	1	0	0	0	0	0	1	0	0	1	68
750	1	6	3	34	24.7				0	0	0	1	0	0	0	6.2
4770	1	10	7	15	21.9	0	1	0	0	0	0	1	0	0	0	65
8760	1	24	30	52	33.5	0	0	1	1	0	0	1	0	0	0	10
	0	9	2	11	21.1				0	0	0	0	0	0	0	4.1

s Foundation of America
otiometry system
nent duration, maximum dose, current dose, 1-year total dose, CNIs: calcineurin inhibitors
D, Ca: calcium, Parents, smoking, PSL 3mo, RA, secondary, alco: risk factors
